# RNA on Display: Cell Surface RNA as a Novel Layer of Cellular Regulation

**DOI:** 10.34133/research.1147

**Published:** 2026-02-27

**Authors:** Serina Chahal, Pious Jose, Adam Greasley, Kevin Vytlingam, Raj Kumar Thapa, Hayley Pyne, Amal Abu Omar, Xiufen Zheng

**Affiliations:** ^1^ Department of Microbiology and Immunology, Western University, London, ON, Canada.; ^2^ Department of Pathology and Laboratory Medicine, Western University, London, ON, Canada.; ^3^ Department of Surgery, Western University, London, ON, Canada.; ^4^ Department of Physiology and Biochemistry, Jordan University of Science and Technology, Irbid, Jordan.; ^5^ The Multi-Organ Transplant Program, London Health Sciences Center Research Institute, London, ON, Canada.; ^6^ Matthew Mailing Center for Translational Transplant Studies, London Health Sciences Center, London, ON, Canada.

## Abstract

RNA has been established as an essential molecule in virtually all cellular processes. Besides its role in cellular modulation via extracellular vesicular release, it has long been thought to only exert its function intracellularly. Recent discoveries have shown evidence of extracellular RNA tethered to the cell surface of different cell types. These cell surface RNAs are predominantly modified with sialylated glycans, denominating them cell surface glycoRNAs. While the expression of cell surface glycoRNAs has been found on a wide array of immune cell and cancer cell types, research on its functional roles has only recently been explored due to challenges in detection, isolation, and sequencing of this new subclass of RNA. In this review, the history, biogenesis, detection and isolation techniques, and functional roles of cell surface RNA to date will be discussed, in addition to commenting on its translational capacity for studying immunomodulation and disease. Acknowledging the presence of cell surface RNAs and propelling our understanding on their function will provide a new avenue to study cellular modulation and cell–cell communication.

## Introduction

The role of ribonucleic acids (RNA) in cellular function is vital. From acting as templates for protein synthesis, regulating gene expression, and catalyzing reactions, RNA has established its role as a key molecule in virtually all cellular processes. Most abundantly, RNA is found in the form of ribosomal RNA (rRNA), where they complex with proteins to form ribosomes, the key machinery involved in protein synthesis. The template for protein synthesis itself is found in the form of messenger RNA (mRNA) that is transcribed from the DNA genome. Transfer RNAs (tRNA) are also involved during protein synthesis and are the builders that bring the correct amino acids to the ribosomes to synthesize a peptide chain. Both rRNA and tRNA also exhibit catalytic activity during protein translation, categorizing them as ribozymes. Additionally, there are naturally occurring regulatory non-coding RNAs (ncRNA), including microRNA (miRNA), long non-coding RNA (lncRNA), and circular RNA (circRNA), which can control gene expression and regulate signal pathways within cells [1].

RNA is traditionally considered to exist within the cell, predominantly in the cytoplasm and nucleus, with some small RNA molecules also being secreted in extracellular vesicles (EVs) [2]. A more recent discovery found that RNA is expressed on the cell surface of many different cell types [3]. These RNAs, called cell surface RNA, are bound to the cell membrane through unknown linkers and are thought to have advanced secondary structure through complementary base pairing interactions [4]. The vast majority of studied cell surface RNAs to date have also been found to be modified with sialylated glycans on their secondary RNA structures, denominating them cell surface glycoRNA [3]. Multiple different names have been used to refer to cell surface RNA in the current literature, including membrane-associated extracellular RNA [5], cell membrane-associated RNA [6], glycoRNA, and surface RNA. Based on the current literature, signs point to all cell surface RNAs being glycosylated; however, some studies have not explicitly stated this due to a lack of detection of glycosylation. It is thus unknown whether the RNA is glycosylated or not in those specific studies, yet there is increasing evidence that all cell surface RNAs are glycosylated [7–9]. For this review, cell surface RNA or cell surface glycoRNA, depending on the scope of the study being examined, will be used interchangeably to describe this novel class of RNAs.

Research on cell surface RNA has only just begun and many groups have implicated them as being a functional component of the cell that is involved in a wide array of cellular activities, predominantly in different immune cell types. In this review on cell surface RNA, we will discuss its history, biogenesis, detection and isolation techniques, functional roles, and comment on its potential application in studying immune dysfunction diseases. The goal of this review is to consolidate current knowledge on cell surface RNA and propel its research and application for future studies in this emerging field.

## Discovery of Cell Membrane RNA

### History of cell membrane RNA

The presence of cell surface-bound RNAs was first described in 2004 by Morozkin et al. [10], where they found binding of extracellularly released RNA on the surface of cancerous cell lines, such as HeLa cells (human cervical carcinoma) and A431 (human squamous cell carcinoma) after long-term culturing [10]. These RNAs were explained to only exhibit transient binding to the cell surface and were easily removable with inert regents such as phosphate buffered saline. In 2010, more stable membrane-associated RNAs were detected in bacteria [11,12], and it was not until 2021 where stable cell surface RNA on cell of human origin were detected by Flynn et al. [3]. They demonstrated that there are glycoRNAs on cell membranes using metabolic labeling and biorthogonal chemistry in vitro and in vivo [3]—precursor sugars were labeled with a clickable azide group, which enabled a biorthogonal reaction with biotin. Then, the biotin–streptavidin system was used to help visualize the cell surface glycoRNA by fluorescent microscopy. They found that majority of cell surface RNAs were glycoRNA and RNA sequencing (RNA-seq) of enriched glycoRNAs helped to identify specific cell surface RNAs. Since their initial detection on human cells by Flynn et al. [3], more cell surface RNAs have been detected in a variety of cells [3–6], and multiple methods have been developed to detect, visualize, and isolate them [13–15]. Several biological functions of cell surface RNA have been determined, most of which are critical cellular roles involved in immunogenic antigen formation, cell surface RNA-binding protein (RBP) stability, cell signaling, communication, and immune cell recruitment.

It is important to note that cell surface RNAs are novel in terms of the discovery of their localization on the cell membrane; however, emerging sequencing data in many studies point toward these RNAs being a part of the existing RNAome of cells, which are then post-transcriptionally modified and selectively trafficked to the cell surface [6]. Prior to trafficking to the cell surface, these pre-existing RNA transcripts are primarily modified with glycan groups through a process known as glycosylation, as discussed next.

### GlycoRNA biogenesis and transport to the cell membrane

Glycosylation is the biochemical process of the addition of sugar chains (glycans) to proteins, lipids, and RNA by a covalent bond [7,16]. It was first discovered almost a century ago when studying differences in blood groups, where researchers found specific glycan blood group antigens [17]. Glycosylation of proteins and lipids has also been known for a long time [18]; however, RNA glycosylation is a more recent discovery [3]. The glycosylation of biological molecules has shown to play a role in protein folding and stability, cell signaling, immune function, cell adhesion, and many other physiological processes [19].

Glycosylation can be of various types, such as O-linked glycans, N-linked glycans, glycosaminoglycans, glycosphingolipids, and phosphorylated glycans [20]. O-glycosylation and N-glycosylation are the most common [21]. N-glycosylation involves attaching N-acetylglucosamine (GlcNAc) to the nitrogen atom of the amino acid Asn, usually found in the Asn-X-Ser/Thr sequence (where X is any amino acid except proline) [22]. O-glycosylation occurs on functional hydroxyl groups, which are often connected with the oxygen atoms on serine and threonine. The most common O-glycosylation type is mucin-type (N-acetylgalactosamine [GalNAc] type) O-glycosylation, which is abundant in mucin glycoproteins found in the mucus and on cell surfaces [23]. So far, sialoglycoRNA (RNA modified with sialylated glycans) and fucoglycoRNA (RNA modified fucose residues) have been detected in human cells [24].

Emerging evidence suggests that the production and surface display of glycoRNAs may be functionally connected to the canonical N-glycosylation pathway. The catalytic subunit of the oligosaccharyltransferase complex (STT3A-OST), which mediates co-translational glycosylation in the endoplasmic reticulum (ER), appears to contribute to this process, as its genetic disruption leads to a pronounced reduction in glycoRNA levels compared to perturbation of its similar STT3B-OST counterpart [9]. Furthermore, experimental manipulation of downstream N-glycan processing factors (including MGAT1, MOGS, and CALR) similarly affects cell surface glycoRNA abundance, potentially indicating a broader dependence on the secretory glycosylation machinery [9]. These observations raise the possibility that cell surface glycoRNA biogenesis could involve vesicular trafficking pathways or utilize N-glycosylated proteins as intermediaries, though the precise mechanistic relationship remains to be fully elucidated.

Although not explicitly identified yet, it would be plausible to assume that there is an intracellular pool of glycoRNA within the secretory pathway (ER/Golgi Appartus) that are then trafficked to the cell surface. There is no evidence in the current literature of RNA glycosylation occurring, or the presence of glycosylation machinery at the cell membrane. Instead, there is evidence of post-translational glycosylation of proteins in the cytoplasm [16,18,19] or secretory pathways, in addition to all other post-transcriptional RNA modifications occurring in the nucleus of cells; thus, it would be plausible to predict the existence of an intracellular pool of glycoRNAs in the cytoplasm, secretory pathways, and perhaps even the nucleus of cells. While the presence of an intracellular pool of glycoRNA most likely is present in cells, current technologies struggle with sensitivity and accuracy when it comes to distinguishing cell surface glycoRNA from intracellular glycoRNA, the latter of which may be present in the secretory pathway or other organelle compartments. Since intracellular and cell surface glycoRNAs are chemically the same, the only difference being their localization, there are only 2 viable strategies to specifically detect cell surface glycoRNA, which include visual detection or specific isolation of the cell membrane. With visual detection, staining for cell surface RNA in conjunction with cell membrane-specific and organelle-specific markers would assist in differentiating between surface-bound and intracellular glycoRNA. With specific isolation of the cell membrane, if a pure cell-membrane fragment is obtained, one could then continue with RNA isolation and sequencing of the cell surface glycoRNAs; however, this method is susceptible to cytoplasmic contamination and provides low RNA yield [25]. The intricacies of how cell surface RNA can be detected, isolated, and sequenced are discussed in detail later in this review.

#### Surface localization of glycoRNAs via Sidt1/2 transporters

Current evidence suggests that the systemic RNA interference-defective-1 transmembrane family member 1/2 (Sidt1/Sidt2) proteins (known mediators of nucleic acid transport from endocytic compartments) may facilitate the surface localization of RNAs [4,24,26,27]. Sidt1 and Sidt2 are homologous, multi-pass transmembrane proteins that act as dedicated RNA transporters in endolysosomal membranes rather than at the plasma surface [4,24,26,27]. Specifically, their multi-pass structure forms a central channel that can selectively bind and translocate RNA molecules across the lipid bilayer to the cell surface. Both proteins are postulated to be required for the presence of sialylated RNAs on the cell surface as it has previously been shown that knockdown of both Sidt1 and Sidt2 in *HOXB8*-differentiated neutrophils completely abolished the detection of cell surface glycoRNAs without affecting the labeling of other sialylated surface components [4]. The Sidt-deficient neutrophils show a >5-fold reduction in in vivo recruitment, phenocopying extracellular RNase treatment of cells, the latter which removes all cell surface RNAs [4]. Sidt1 and Sidt2 were also shown to interact physically (detected by FRET–FLIM) and to colocalize in late endosomes/lysosomes, suggesting a cooperative transport module [26]. They likely function as a heterodimeric complex, increasing transport efficiency and RNA specificity compared to either protein alone. This potential glycoRNA trafficking mechanism appears distinct from RNA–EV secretion pathways, as suggested by the dependence on Sidt transporters rather than EV export machinery [4,26]. This alternative trafficking mechanism may also explain the RNase sensitivity of surface-localized glycoRNAs, as they are not protected in EVs, and the similar functional defects observed in both Sidt1/2-knockdown cells and RNase-treated wild-type neutrophils, including reduced adhesion and impaired transendothelial migration [4,26].

**Fig. 1.**
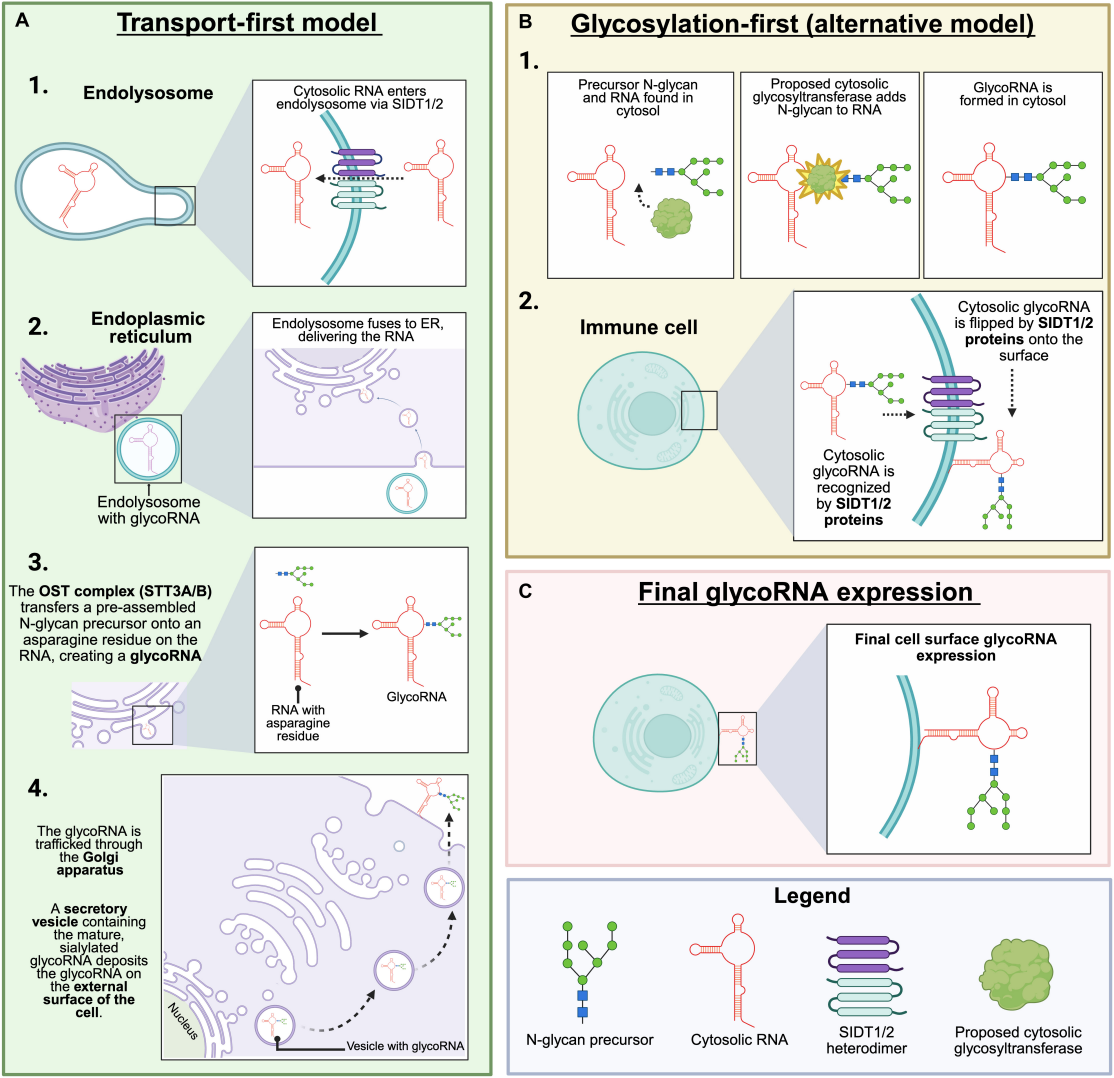
Models for Sidt1/2-dependent glycoRNA biogenesis and surface display. (A) In the “Transport-first” model, Sidt1/2 heterodimers in endolysosomal membranes import cytosolic RNA into the lumen of the secretory pathway. The RNA is glycosylated by the luminal OST complex, processed in the Golgi, and delivered to the cell surface via exocytosis. (B) In the “Glycosylation-first” model, RNA is glycosylated by an unknown cytosolic enzyme before Sidt1/2 complexes, potentially at the plasma membrane, “flip” the intact glycoRNA to the outer portion of the membrane. (C) Both models from (A) and (B) result in cell surface glycoRNA expression. Created in BioRender. Jose, P. [2026] https://BioRender.com/h7oggga. OST complex (STT3A/B), catalytic subunit of the oligosaccharyltransferase complex; Sidt1/2, systemic RNA interference-defective-1 transmembrane family member 1/2.

Building on this evidence, there are 2 plausible mechanisms of action for Sidt-mediated glycoRNA transport to the cell surface (Fig. 1). In the “Transport first” model, cytosolic RNAs are imported by Sidt1/2 into the lumen of an ER/Golgi compatible compartment, become N-glycosylated by the canonical OST complex (STT3A/B), and are then secreted via the conventional secretory pathway to the cell surface [4,27,28]. Here, Sidt1/2 act as the critical gatekeepers, moving RNA into the secretory pathway for the first time, resulting in glycoRNA that is then delivered to the cell surface via vesicular trafficking. The alternative “Glycosylation first” model posits that RNAs acquire N-glycans in the cytosol (or at the cytoplasmic face of the ER) before Sidt1/2 “flip” or escort them across the plasma membrane. The intracellular localization of Sidt proteins in endolysosomes makes the former model more plausible [4,27,28]. This is because the endolysosomal system is a central hub that intersects with the secretory pathway, providing a perfect conduit for RNAs to be delivered into organelles where glycosylation occurs, before being routed to the surface via vesicles. Current detection methods (metabolic labeling with Ac₄ManNAz [azide-containing monosaccharide], proximity ligation assay [PLA], and extracellular RNase A digestion) preferentially capture RNAs that have traversed the secretory route and are exposed on the outer cell surface; intracellular intermediates remain invisible unless organelle-specific fractionation is performed [4]. Thus, the exact mechanism by which Sidt 1/2 select, load, and release specific RNA cargo remains a key open question. A critical and unresolved question is whether Sidt1 and Sidt2 have redundant functions or distinct roles in this process. Identifying the specific sequences or structural features that designate certain RNAs as cargo for glycosylation and transport will be essential to fully elucidate this novel biosynthetic pathway.

As for the expression of Sidt proteins, in mice, Sidt1 is highly expressed in the brain, thymus, T cells, NK cells, plasmacytoid dendritic cells, and neutrophils, with modest expression levels in B cells and other hematopoietic cells [24]. Human datasets show similar enrichment in immune subsets and strong expression in gastric epithelial cells. Many cancer cells also show an up-regulation of Sidt1, which correlates with advanced stage and poorer survival, as seen in non-small cell lung cancer (NSCLC) (especially squamous carcinoma), breast cancer, pancreatic cancer, and gastric cancer [29]. For example, several epithelial cancer lines (e.g., NCI-H441) exhibit the highest surface glycoRNA levels, likely reflecting their robust N-glycosylation machinery [24]. By comparison, many non-immune tissues exhibit low or undetectable Sidt transcripts, suggesting that only Sidt-positive cells can efficiently surface-display glycoRNAs [24]. Sidt1 likely facilitates the transport of these RNAs to the cell surface. Since many normal epithelial tissues have low or undetectable Sidt expression, the cancer-specific up-regulation of Sidt1 drives an altered, tumor-associated glycoRNA landscape that may contribute to tumor progression, chemoresistance, and, ultimately, poorer clinical outcomes [29].

Despite these recent discoveries, the full transport pathway and machinery involved in cell surface RNA biogenesis, glycosylation, and trafficking requires further work. Additionally, determining the mechanism or criteria by which specific RNAs are selected for cell surface presentation is an important avenue to discover for continued work in this field as it will help provide insight not only on the biosynthetic pathway, but also on the cellular distribution and physiological role of cell surface RNAs.

## Cell-type distribution of cell surface RNA

The distribution of cell surface RNA spans across a diverse array of cell types, including both prokaryotic and eukaryotic cells (Table 1). In the current literature, cancerous cells are the most studied cell type in terms of cell surface RNA detection, followed by innate immune cells and finally non-cancerous, non-immune somatic cells. From all 3 cell type categories described previously, those of human origin are the most studied; however, albeit less studied, cell surface RNAs have additionally been detected on cells of mouse and hamster origin. The current distribution of cell surface RNA presently found in the literature could simply be attributed to researchers studying cells where surface RNAs could potentially have the biggest impact on health and disease (immune dysfunction diseases), such as serving as biomarkers for cancer or targets for novel therapeutics against immune-related diseases. It is most likely that all somatic cells express cell surface RNA, yet whether they have any implication on the function of non-immune cells, their associated diseases, or as biomarkers for non-neoplasm diseases needs to be further elucidated.

**Table 1.** Summary of the cellular distribution of cell surface RNAs.

Cell type	Cell source	Glycosylation state	Species/Organism	Relative expression level (within specific study)^a^	Source
Cervical carcinoma	HeLa	Positive	Human		Flynn et al. [3]
					Li et al. [15]
					Ma et al. [14]
Squamous cell carcinoma	A431	Unknown	Human		Morozkin et al. [10]
Gram-positive bacteria	*B. halodurans*	Unknown	Bacteria		Block et al. [12]
Melanoma	Mel-525	Positive	Human		Li et al. [15]
Osteosarcoma	U2-OS	Positive	Human		Li et al. [15]
Ovarian cells	Chinese Hamster Ovary	Positive	Hamster		Li et al. [15]
Non-small cell lung cancer	A549	Positive	Human		Abledu et al. [24]
					Perr et al. [31]
Acute myeloid leukemia	AML3	Positive	Human		Perr et al. [31]
Chronic myeloid leukemia	K562	Positive	Human		Perr et al. [31]
					Flynn et al. [3]
Embryonic kidney cells	HEK293	Positive	Human		Perr et al. [31]
					Flynn et al. [3]
Lung adenocarcinoma	NCI-H441	Positive	Human	+++	Abledu et al. [24]
Immortalized alveolar epithelial cells	hAELVi	Positive	Human	+	Abledu et al. [24]
Primary alveolar epithelial cells	hPAEpC	Positive	Human	+	Abledu et al. [24]
Neutrophils	Immortalized neutrophils	Positive	Mouse		Zhang et al. [4]
Neutrophils	PMN	Positive	Mouse		Zhang et al. [4]
Mature neutrophil-like cells	HL-60	Positive	Human		Ma et al. [14]
Malignant breast cancer	MCF-7	Positive/Negative	Human	++	Ma et al. [14]
					Lv et al. [6]
Non-malignant breast epithelial cells	MCF-10A	Positive/Negative	Human	+++	Ma et al. [14]
					Lv et al. [6]
Metastatic breast cancer	MDA-MB-231	Positive/Negative	Human	+	Ma et al. [14]
					Lv et al. [6]
Malignant breast cancer	SK-BR-3	Unknown	Human		Lv et al. [6]
Glioblastoma	L299/U87	Positive	Human		Xin et al. [28]
Monocytes	THP-1	Positive	Human	++	Ma et al. [14]
M0 macrophages	THP-1	Positive	Human	+	Ma et al. [14]
LPS-activated M0 macrophages	THP-1	Positive	Human	+++	Ma et al. [14]
Macrophage	RAW 264.7	Positive	Mouse		Graziano et al. [36]
T lymphoblast	EL4	Unknown	Mouse	+++	Huang et al. [5]
CD14^+^ monocyte	PBMC	Unknown	Human	+++	Huang et al. [5]
Dendritic cells	PBMC	Unknown	Human	++	Huang et al. [5]
CD3e^+^ T cells	PBMC	Unknown	Human	+	Huang et al. [5]

^a^ Comparison of relative expression levels of cell surface RNAs between cell types was only possible within studies that directly compared expression levels

### Cell surface RNAs on bacteria

The detection of more stable cell surface RNAs began with bacterial studies of extremophile gram-positive bacteria such as *Bacillus halodurans* [11,12]. Block et al. [12] found that large, non-coding RNAs called ornate, large, extremophilic (OLE) RNAs preferentially formed ribonucleoprotein complexes with OLE-associated protein and were localized to the cell membrane in *B. halodurans*. A more recent study by Breaker et al. [30] has showcased that altering these OLE RNAs resulted in reduced gram-positive bacterial cell growth in different stress environments, indicating that these cell surface RNAs play a role in cell signaling and are essential for bacterial adaptation and survival.

### Cell surface RNAs on tumor cells

Cell surface glycoRNAs have been identified on multiple human cancer cell lines, including melanoma, osteosarcoma, cervical carcinoma, lung cancers, acute myeloid leukemia, chronic myeloid leukemia, and breast cancers (malignant and non-malignant) (Table 1). Colocalization of cell surface RNA with RBPs on cancer cells has also been demonstrated in a more recent study by Perr et al. [31]. They found that treatment of cells with RNase disrupted the clustering and presentation of RBPs on the cell surface of many different human cancer cell types, including NSCLC (A549), acute myeloid leukemia (AML3), chronic myeloid leukemia (K562), and embryonic kidney cells (HEK293), indirectly indicating that cell surface RNAs are not only present on these cell types, but also colocalized with RBPs. From the current literature, the expression of cell surface glycoRNA in human cancers seems to follow different trends depending on tissue origin and stage of cancer progression.

#### Expression of cell surface RNA in breast cancer cell lines

One study detected the presence of cell surface glycoRNAs using in situ hybridization (ISH) fluorescent imaging on many different human breast cancer cell types, including MCF-7 (malignant breast cancer), MCF-10A (non-malignant breast epithelial cells), and MDA-MB-231 (metastatic breast cancer) [14]. Within the repertoire of breast cancer cell lines examined, non-carcinomatous cells had the highest cell surface glycoRNA abundance, followed by highly malignant cells and then metastatic breast cancer, indicating that the expression of cell surface glycoRNA follows different trends in cells depending on the state of cancer progression.

A study from Lv et al. [6] detected cell surface RNA using MREMB (Membrane-associated RNA Extraction based on Magnetic Beads) in breast cancer cell lines (MCF-7, MDA-MB-231, and SKBR-3) and a non-neoplastic breast epithelial cell line (MCF-10A) [6]. By analyzing differentially expressed cell surface RNAs between breast epithelial cells and breast cancer cells using a Gene Ontology database, researchers found that breast cancer cell lines have enriched cell surface RNAs with functions involved in the extracellular matrix (ECM) regulation, cargo receptor activity, gated channel activity, cell adhesion, and multicellular organism development. Additionally, using RNA-seq, researchers found that differences in cell surface RNA composition among non-neoplastic and neoplastic breast cancer cell lines are mainly reflected in the ECM [6]. Specifically, 128 cell surface RNAs related to the ECM, including genes specifically associated with collagen (SPON2, MUC4, COL12A1, and CNN1), were up-regulated in neoplastic breast cancer cell lines compared to MCF-10A cells. Additionally, among the enriched lncRNAs detected on the cell surface, 268 showed differential expression when comparing neoplastic breast cancer cells to non-neoplastic breast cancer cells. The detection of membrane-associated mRNAs and lncRNAs by Lv et al. [6] challenges the results in existing literature that cell surface RNAs are predominantly small RNAs [3]. A study by Flynn et al. [3] found that when separating RNA based on size, glycoRNAs were only found in the small RNA (<200 nt) population, indicating that larger RNA molecules may not be glycosylated. A plausible distinction between the two is that RNAs that are post-transcriptionally modified with glycan groups and then trafficked to the surface are indeed small RNAs and what we know as cell surface glycoRNAs, while lncRNAs or mRNAs on the surface are most likely non-glycosylated [6]. Further, there is also speculation that lncRNAs or mRNAs found on the cell membrane may not be true cell surface RNAs and are instead just an intermediary state of RNA molecules that are destined to be secreted. Previous studies have shown evidence that both lncRNA and mRNA can be secreted by cells within vesicles or “freely” secreted bound to RBPs [32–34], and thus further investigation into the purpose of these long RNAs found on the cell membrane is required. As research on cell surface RNAs increases over the next decade, sequencing data on cell surface RNAs will be vaster and will allow for conclusions to be made on what the true nature of cell surface RNAs are, in terms of size, glycosylation state, and protein-coding capacity.

#### Expression of cell surface RNA in lung cancer

The majority of identified cell surface RNAs were found to be modified via glycosylation, and the expression of cell surface glycoRNAs varies based on both the cell type and glycosyl group modification being examined. Abledu et al. [24] detected the expression of cell surface glycoRNA across different human alveolar epithelial cell lines, including lung adenocarcinoma (NCI-H441), NSCLC (A549), immortalized human alveolar epithelial cells (hAELVi), and primary alveolar epithelial cells (hPAEpC) using metabolic labeling of glycosyl groups [24]. Expression levels of sialoglycoRNA were found as follows: NCI-H441 > hPAEpC > hAELVi > A549, while the expression of de novo formed fucoglycoRNA was as follows: NCI-H441 > A549 > hPAEpC > hAELVi. Moreover, the NCI-H441 cell line had the largest abundance of total glycoRNAs, regardless of the type of glycoRNA examined. It is hypothesized that the differences in glycoRNA abundance between cell types may be associated with barrier integrity, with those that form tight epithelial monolayers (NCI-H441) having increased glycoRNAs, while those with lower barrier function (A549) have a relatively lower abundance of glycoRNAs [24]. It is also clear that the non-cancerous cell types examined consistently had lower levels of cell surface glycoRNAs relative to lung adenocarcinoma, while their expression relative to NSCLC was dependent on the type of glycoRNA examined.

#### Expression of cell surface RNA in other cancer types

Cell surface RNA present on the surface of multiple human cancer cell lines (melanoma [Mel-525], osteosarcoma [U2-OS], and cervical carcinoma [HeLa]) was preferentially found to be associated with heparan sulfate (HS), a glycosaminoglycan that is found on the plasma membrane and ECM, and is involved in ligand docking on cells [15]. Li et al. [15] found that the knockout of HS in Chinese hamster ovary cell mutants resulted in a loss of detection of cell surface RNA via toll-like receptor 7 (TLR7) (binds ssRNA), indicating that HS may be needed for the presentation of RNA on the cell surface [15]. They also found HS-associated cell surface RNA to be associated with multiple RBPs, which were formerly thought to be only found in intracellular locations. They further revealed that HS, cell surface RNA, and RBPs are all found in proximity to one another, with the latter two requiring HS to be stably expressed on the cell surface. Further, Li et al. [15] identified genes essential for stable cell surface RNA expression using pooled genome-wide CRISPR-Cas9 knockout screening [15]. Again, using TLR7 as an identifier of cell surface RNAs, a cell surface RNA “lo” and “high” phenotype was measured using flow cytometry and the targeted genes knocked out to produce either phenotype were identified via RNA-seq. These results revealed that knockout of enzymes involved in HS regulation, both related to glycosylation and HS biosynthesis, were associated with a “lo” surface RNA phenotype, indicating these enzymes are important for surface RNA expression [28].

Evidence of cell surface glycoRNA expression has also been found on human glioma cell lines (U87 and LN229) [28]. Through metabolic labeling of glycoRNA and click chemistry [3,24], Xin et al. [28] found that glioma cells had very high expression of cell surface glycoRNAs and implicated them in controlling glioma proliferation and viability.

#### Expression of cell surface RNA in murine cancers

In addition to human cancer cells, Huang et al. [5] detected and sequenced cell surface RNAs on murine T cell lymphoblast cells (EL4) to optimize their Surface-Seq and Surface-fluorescence in situ hybridization (FISH) techniques, confirming the presence of nuclear-encoded RNAs, including MALAT1 and NEAT1, on the surface of live and intact cells [5].

### Cell surface RNAs on immune cells

#### Expression of cell surface RNAs on human immune cells

Cell surface RNA expression was first identified on immune cells during a clinical sample-based study by Laktionov et al. [35], where they found that extracellular nucleic acids, including both RNA and DNA, were differentially found in the blood of healthy donors versus patients with breast tumors (both malignant and non-malignant) [35]. Almost all of the nucleic acids detected in healthy donors were found bound to the surface of blood cells, including both erythrocytes and leukocytes, while in cancer patients, they were instead largely found in the blood plasma, indicating surface nucleic acid shedding.

GlycoRNAs have also been detected in THP-1 monocytes and THP-1-derived macrophages, including M0 macrophages and LPS-activated M0 macrophages (or M1 macrophages) [14]. One study found that the relative levels of glycoRNA differed depending on the activation state of macrophages, with levels decreasing after differentiation from monocyte to M0 macrophage and then increasing in inflammatory macrophages after stimulation with LPS.

Apart from immune cell lines, cell surface RNAs have been detected on immune cells in the peripheral blood and bone marrow. One study used fluorescently labeled oligonucleotide probes (in situ surface FISH [isFISH]), followed by imaging flow cytometry analysis, to detect cell surface RNA on human peripheral blood mononuclear cells (PBMCs) [5]. Huang et al. [5] found that, on average, 4.8% of total human PBMCs were isFISH^+^ (cell surface RNA positive), with over 10% of CD14^+^ monocytes and approximately 3% of CD3ε^+^ T cell populations having positive isFISH signals compared to <2% of CD19^+^ (B cells) and CD3ε^−^CD14^−^CD19^-^ cells (other immune cells). Additionally, single-cell transcriptomic analysis revealed that isFISH^+^ cells were enriched in monocyte markers (CD14 and KLYS) while isFISH^−^ clustered cells were enriched in T cell, NK cell, and B cell markers (CD3E, CD8A, NKG7, and MS4A1). Additionally, using the SingleCellNet classifier, isFISH^+^ populations were further classified into CD14^+^ monocytes and dendritic cells, with monocytes accounting for 87% of the total number of isFISH^+^ cells [5]. These findings show that cell surface RNAs are not uniformly present in all immune cell types, with monocytes being the major cell surface RNA-presenting cell type in human PBMCs.

#### Expression of cell surface RNAs on murine immune cells

Another study found the expression of cell surface glycoRNA on both immortalized neutrophils created via in vitro differentiation of murine bone marrow progenitor cells and primary neutrophils (PMNs) purified from murine bone marrow [4]. They also indirectly showed the colocalization of cell surface glycoRNAs with proteins on the neutrophil surface as the detection of the cell surface RNAs or their removal with RNase A digestion was not possible without prior proteinase K treatment of the cells to remove any surface proteins. The working theory is that cell surface proteins “protect” the glycoRNA from degradation in the extracellular environment [4].

A more recent study by Graziano et al. [36] also found expression of cell surface glycoRNAs on murine macrophages (RAW 264.7), distinguishing that these surface RNAs were glycosylated with N-glycans at the modified RNA base acp^3^U.

## Methods for Studying Cell Surface RNA

The study of cell surface RNA has sparked the innovation of numerous protocols, each with the goal of isolating, sequencing, or visualizing RNA exclusively on the cell surface. Each method has its advantages and limitations, but can each provide important insight into the function of cell surface RNA.

### Isolation and sequencing of cell surface RNA

#### Isolation techniques for cell surface RNA

The isolation of surface RNA has predominantly been approached with the goal of sequencing the isolated RNAs. Multiple isolation techniques have been used, but they both rely on 2 main principles: (a) that cell surface RNAs are all anchored in the cell membrane and (b) glycoRNAs are predominantly present on the cell membrane and are not cytoplasmic.

Originally, the enrichment of cell surface RNA was attempted through the isolation of large, intact fragments of the cell membrane using a series of lysis buffers and ultracentrifugation [37]. This technique yielded reasonably enriched membrane fractions with detectable RNA; however, the quantity and quality of RNA extracted was challenging for downstream analysis, such as sequencing, due to low input, longer protocol times, and subpar quality. More recently, efforts to improve this enrichment using nanobeads have been undertaken. Cationic magnetic-nanobeads (C-MB) capable of attaching to live cells and maintaining an envelope around the membranes post-disruption are used to create enriched fractions containing cell membranes [6]. C-MB isolation coupled with hydroxyl magnetic-nanobeads (H-MB) to recover RNA from the C-MB fractions yielded a much higher quantity of membrane-bound proteins and a higher yield of RNA with reasonable quality for sequencing compared to previous ultracentrifugation workflows [6]. An alternative approach using nanobeads was also undertaken by Lv et al. [6], where instead of coating membranes for isolation, they coated polymeric cores with cell membranes, allowing for the naïve orientation of the membranes to remain intact, which they dubbed membrane-coated nanoparticles (MCNPs).

The second major method used for the isolation of cell surface RNAs comes from the use of click chemistry [3,24]. This method relies heavily on the 2 principles above, as well as the assumption that the majority of cell surface RNAs are glycosylated. In this scenario, cells are fed non-natural azide variants of the sialic acid precursor such as N-acetylmannosamine or the fucose precursor L-fucose. In this case, cells were fed N-azidoacetyl-mannosamine (Ac4ManNAz) and 6-azido-L-fucose (FucAz) for 24 h and then conjugated to dibenzocyclooctyne-PEG4-Biotin (DBCO-Biotin). Protocols have utilized click chemistry coupled with streptavidin to isolate cell surface glycoRNA from total RNA extracts or from previously labeled surface RNA, ensuring minimal cytosolic contamination [3,24].

Although not currently applied, a potential third method to isolate cell surface RNA is to label only cell surface-exposed RNA with biotin. Live cells treated with a membrane-impermeable, amine-reactive biotinylating reagent would have only exterior RNA labeled. Biotin-tagged surface RNAs can then be isolated from other cell components containing non-biotinylated RNA. Similar processes have previously been successful in isolating cell surface proteins [38] and could prove useful to isolate cell surface RNAs. Care must be taken to carry out this process as cell surface proteins and DNA could also be tagged and isolated, requiring treatment of cells with DNases and proteinases to remove these confounding molecules.

#### Sequencing of cell surface RNA

Sequencing of cell surface RNA has largely followed standard next-generation sequencing (NGS) protocols using small library preparation and standard NGS workflows to identify any RNA present [6]. One key component of sequencing is the lack of RNA processing. Unlike most NGS workflows, which typically use rRNA depletion pre-library preparation, when sequencing cell surface RNA it is important to use the total isolation (no depletion) without further processing to ensure all sequences are captured. One major disadvantage of NGS with respect to cell surface RNA is the lack of spatial data. The orientation by which the RNA is anchored to the membrane and the regions of RNA exposed to the extracellular environment are important considerations that cannot be answered with standard NGS. Therefore, Huang et al. [5] developed Surface-seq, a methodology to overcome this issue and provide insight into anchoring orientation. Utilizing the MCNPs mentioned previously, they ligated an adapter to the 3′ end of extracellular-facing RNA strands. Comparative analysis of NGS and surface-seq between the same samples shows that surface-seq provides unique outputs compared to standard NGS [5]. This suggests that orientation of cell surface RNA-seq could play a crucial role to validating which targets are unique to the surface and which end of the RNA is freely exposed to the extracellular environment; the latter also provides information on possible glycosylation sites.

### Detection and visualization of cell surface RNA

**Fig. 2.**
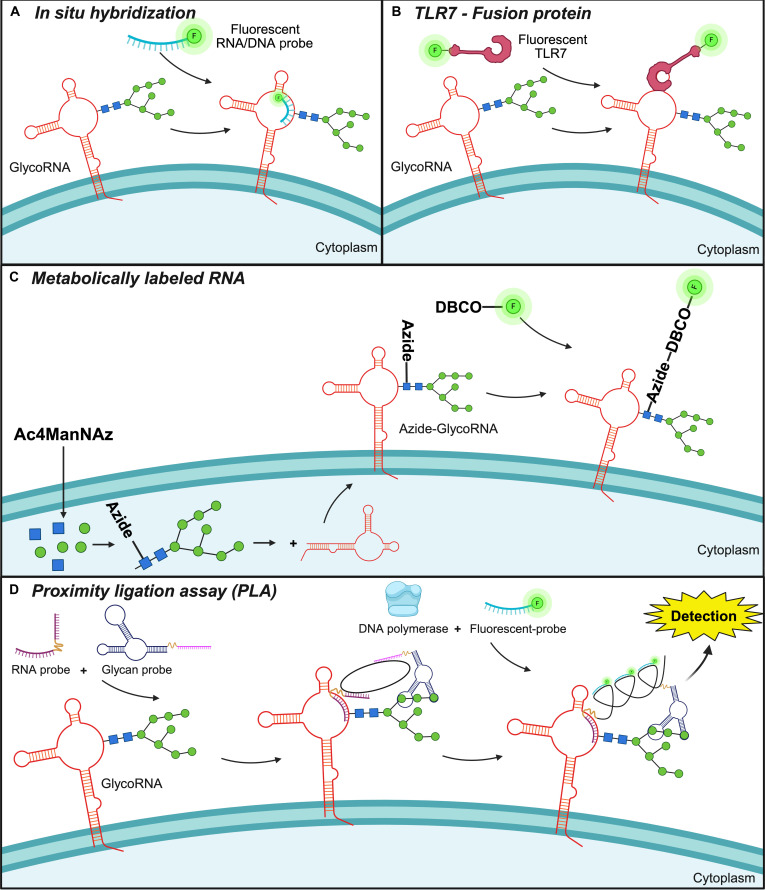
Summary of methodology used to visualize cell surface RNA. (A) In situ hybridization, to detect naïve surface RNA using RNA or DNA based probes. (B) TLR7-Fusion protein, to detect single-stranded cell surface RNA, coupled to a fluorophore for visualization. (C) Metabolically labeled RNA, using amine-reactive monosaccharides to detect surface glycoRNA. (D) Proximity ligation assay (PLA), dependent on binding to both the RNA and the glycan group, followed by PCR to amplify the signal and subsequent binding of probes. Created in BioRender. Greasley, A. [2026] https://BioRender.com/92rqxw3. TLR7, toll-like receptor 7; glycoRNA, glycosylated RNA; Ac4ManNAz, N-azidoacetylmannosamine-tetraacylated.

In the current literature, several different avenues have been explored to detect and visualize cell surface RNA, all with their own advantages and disadvantages depending on the desired experimental output. A summary of the methodology used to visualize cell surface RNA can be found in Fig. 2.

#### RNA FISH

Visualization of cell surface RNA has been achieved using several methods, including traditional methods such as RNA FISH. Specifically, RNA FISH employs fluorescently labeled RNA oligonucleotide probes to hybridize with cell surface RNAs. This technique primarily utilizes probes targeting known RNAs such as the lncRNAs Malat1 and Neat1 [5]. However, FISH requires specific sequence knowledge and lacks the ability to (a) quantify total changes in cell surface RNA, and (b) amplify weak hybridization signals, the latter of which is vital as there is low expression of these RNAs on the cell surface. Therefore, more robust methods that allow for total, quantifiable detection and stronger signal amplification have been developed.

#### Utilization of metabolically labeled RNA

The metabolic labeling of RNA is not solely utilized for its isolation, but it has also been used for visualization. In these scenarios, the same principle of using FucAz or AC4ManAz has been used to label cell surface glycoRNA, where they are then conjugated with DBCO tagged with a fluorophore instead of biotin [3,24]. The major advantage to this model is that it can be done with live cells that require no manipulation, meaning that live-cell confocal imaging can be achieved. Alternatively, 5-bromo-2-uridine (BrU) has been used to metabolically label cell surface RNA [4]. Following BrU, a biotinylated-anti-BrdU antibody is used to detect surface RNA via microscopy. Since BrU labels all RNA associated with a cell, both intracellular and extracellular, care needs to be taken to ensure the cells are not permeabilized during antibody labeling. This step ensures specificity to cell surface RNA; however, additional steps such as proteinase K digestion is needed to allow antibody access to the RNA, which aligns with previous studies that have indicated that surface RBPs may “protect” or hinder access to cell surface RNAs [4]. Although the BrU method allows for more specific RNA detection, the increased number of steps, handling, and treatment of cells could permeabilize the cells and lead to false positives; therefore, an abundance of controls (i.e., permeabilized cells, cell compartment staining, etc.) and labor are needed to validate the accuracy of the staining.

#### Detection of native cell surface RNA

Although metabolic labeling provides an easy and effective method to visualize RNA in general, it does result in cell surface RNAs being modified from their native state to allow for detection. Therefore, methods that can detect native cell surface RNAs are needed. Traditional methods such as FISH have proven effective if specific RNA sequences have been identified on the cell surface, but it lacks the robustness to visualize total surface RNA, where most sequences are unknown. Two methods that have stood out to accomplish this are the proximity ligation assay (PLA) [14] and a TLR7 fusion protein detector (TLR7-Fc) [15].

PLA requires recognition of 2 moieties that could include DNA, RNA, or protein. Probes for these targets contain a free tail that is complementary to a linker. Should both targets be in proximity, the linker is able to effectively bind both tails. Probes for both targets are bound and coupled with a ligase reaction and subsequent PCR to amplify the signal to indicate positive binding. To apply this to cell surface RNA, Ma et al. [14] developed a PLA that recognizes the sialic acid of glycoRNA, as most surface RNAs were found to be enriched in sialylated structures and sialic acid residues, a method that they denominated: sialic acid aptamer and RNA ISH-mediated proximity ligation assay (ARPLA) [14]. For ARPLA, a glycan probe that recognized the N-acetylneuraminic acid (Neu5Ac) of the glycan was successfully used to detect small nuclear RNA U1 on the cell surface. They were able to use ARPLA to show changes in surface RNA expression in THP-1 cells and show colocalization with proteins [14]. This integrated approach enabled high spatial resolution visualization of surface RNAs; however, the signal in this technique remains weak, and it lacks hybridization with a specific sequence for surface RNAs, which has yet to be discovered. Liu et al. [39] employed a similar technique using a second-generation hierarchical coding strategy (HieCo2) to visualize cell surface glycoRNAs. In HieCo2, DNA codes were covalently attached to sialic acids, then linked to target RNAs via sequence-specific hybridization [39]. This was followed by a series of decoding steps and signal amplification specific to the sialic acid residues of cell surface glycoRNAs.

The TLR7-Fc method was developed by Li et al. [15] with the purpose of detecting total, cell surface RNA. They found that if you use the ectodomain of the TLR7 protein, Ala27–Asn839, this was sufficient to recognize a double uridine motif without other structural or sequence requirements, allowing for easy detection of exposed RNA on the cell surface [15]. This TLR7 epitope was then fused with a human Fc epitope allowing for anti-human IgG fluorescent secondary antibodies to be used for detection. This method follows standard immunocytochemistry workflows and offers a unique, yet simple tool for visualizing cell surface RNA. A recent study by Graziano et al. [36] further confirmed that TLR7, as well as TLR3, can detect cell surface RNAs as primary bone marrow-derived macrophages (BMDMs) isolated from mice lacking either TLR7 or TLR3 adapters did not elicit macrophage activation in response to de-N-glycosylated cell surface RNAs, as determined by a reduction in Type I interferon production by the macrophages.

### Challenges and limitations with cell surface RNA methodology

During this review, we discussed multiple methods for detecting, visualizing, and assessing cell surface RNA. Each method has its advantages and limitations and can answer different research questions. Therefore, prior to selecting a method, it is important to understand how the application of each methodology can affect the desired outcome. A summary of each methodology and their associated specifications can be found in Table 2.

**Table 2.** Summary of methodologies for the study of cell surface RNA.

Category	Methodology	Output from methodology	Advantages	Limitations
Isolation	Cell membrane isolation magnetic beads	Intact cell membrane fractions containing cell surface RNA	Robust and compatible with most cell lines	Low RNA purity that requires more processing
				Unspecific
				Low sensitivity
	Metabolically labeled surface RNA	Isolation of all glyco-RNA from cells	Robust and compatible with most cell lines	Requires RNA turn-over following treatment
				Unspecific
				Low sensitivity
	Biotinylation of live cells	Isolation of accessible cell surface RNA	Provides direct labeling of currently expressed surface RNA on live cells	Not practiced or used much so success rate is unknown
Sequencing	Total RNA sequencing	RNA sequences	High throughput	Lacks spatial orientation of RNA in membrane
			Detects all RNA sequences present	
	Surface-Seq	Surface RNA sequences including orientation	High sensitivity and provides spatial data	Low throughput
				Complex and expensive without industrial support
Visualization	In situ hybridization	Fluorescent imaging	Highly specific	Lower sensitivity and signal
				Low throughput
	Metabolically labeled surface RNA	Fluorescent imaging	Strong signal usually	Nonspecific
				Can lead to high background noise and unspecific binding
	TLR7-Fc	Fluorescent imaging	Detects naïve surface RNA on live cells	Complicated to produce
	Proximity ligation assay	Fluorescent imaging	Highly specific and sensitive	Expensive
				Low throughput
	Electron microscopy	High-resolution images	High resolution for colocalization	Extremely low throughput

#### Challenges of isolation and sequencing

There are multiple proven methodologies for the isolation and sequencing of cell surface RNA, which all in turn translate to a higher success rate regardless of whether ultracentrifugation, magnetic bead membrane isolation, or metabolic labeling is used. The major consideration when isolating surface RNA is to consider the downstream applications of the experiment. If the goal is isolation of all surface RNAs, for sequencing or profiling, then whole membrane isolation coupled with total RNA-seq is a fast, robust, and reliable pipeline. However, if measuring changes of a specific surface RNA molecule is of interest, total membrane isolation may lead to high background and low sensitivity. Therefore, a more specific approach, such as click-chemistry and/or membrane isolation followed by RNA purification, whether generic or targeted for a sequence of interest, would allow for detection of the target with greater sensitivity.

Sequencing of surface RNA faces less challenges due to it being compatible with standard NGS workflows and sample preparation. The only limitation of NGS in the context of surface RNA is that it does not provide any information on membrane anchoring orientation; it cannot discern what end of the RNA molecule is anchored to the surface and how much RNA is exposed to the external environment versus embedded in the membrane, both of which are important to ascertain the function of cell surface RNA. Surface-seq overcomes this, but introduces new limitations such as low throughput, high costs, and a more challenging sample preparation series with no industrial support.

#### Challenges of visualizing surface RNA

Multiple methods for visualizing cell surface RNA have proven effective, but they each have their own distinct limitations. Visualizing total surface RNA has the advantages of seeing global cell surface RNA expression, but often requires metabolically labeled RNA or fixed cells, meaning that global changes in naïve surface RNA expression are challenging to quantify. In addition, they offer a higher signal than single-stranded RNA probes, but cannot detect changes in surface RNA unless they are globally knocked-out. Specific probes such as ARPLA or standard ISH offer high sensitivity and produce strong enough signals to visualize, but the protocols are laborious and low throughput, meaning higher sample volumes are required to generate effective visual signals, the latter of which itself can be difficult to obtain [14].

In addition to visualizing the surface RNA, there are doubts about the ability of fluorescent detection for colocalization of surface RNA with proteins. Although Ma et al. [14] performed brief colocalization with cell surface RNA and protein, Huang et al. [5] performed a more in-depth analysis and found that even super-resolution microscopy lacks the ability to resolve true colocalization of cell surface RNA–protein complexes. They suggested that only electron microscopy could resolve these structures sufficiently; however, the spareness of these structures along the cell surface requires immense amount of scanning and would render electron microscopy impractical as a standard tool unless specific structures or areas of the cell membrane were to be observed. Therefore, although detection and visualization of cell surface RNA, whether global or specific, can be achieved, colocalization of surface RNA–protein complexes is yet to be sufficiently detected or proven.

## Biological functions of cell surface RNAs

**Fig. 3.**
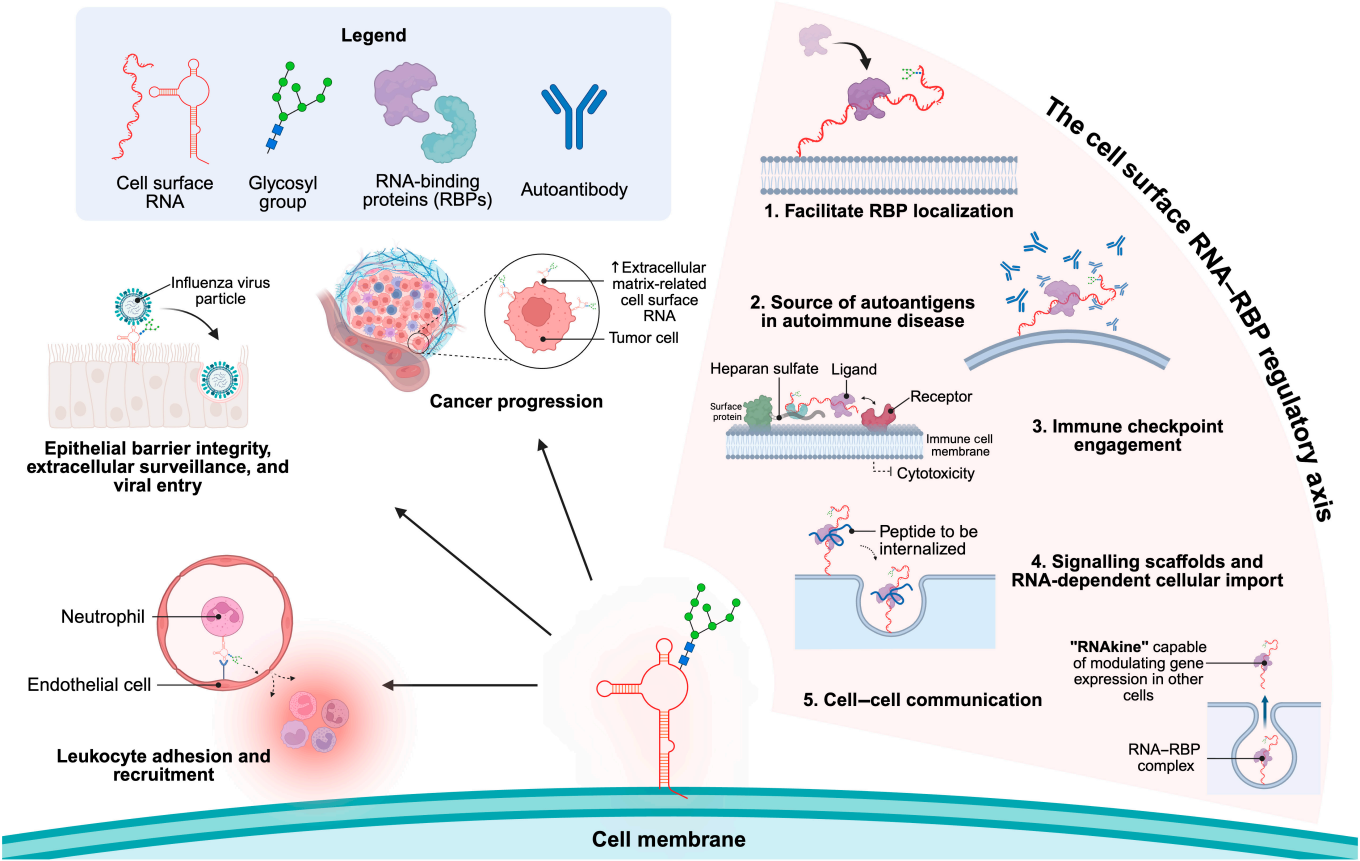
Summary of physiological and pathological roles of cell surface RNAs. Cell surface RNAs have been particularly well-characterized in immunomodulation, making them novel molecules of interest to study immune-related diseases and serve as targets for therapeutic applications. Cell surface RNAs have also been shown to regulate the localization of RNA-binding proteins (RBPs). When complexed with RBP, cell surface RNAs may act as a source of autoantigens in autoimmune disease, engage immune checkpoints, serve as signaling scaffolds, and facilitate communication between cells. Created in BioRender. Vytlingam, K. [2026] https://BioRender.com/jz7vkmo.

Given that the paradigm-shifting discovery of RNA species existing extracellularly on the cell membrane is fairly recent, biological functions of cell surface RNAs remain largely unexplored. Emerging functions of cell surface RNAs implicate them in cell-to-cell interactions, immune modulation, epithelial cell integrity, and the pathogenesis of disease (Fig. 3).

### Functions of cell surface RNAs on immune cells

#### Facilitators of leukocyte–endothelial adhesion and immune cell recruitment

Mediating leukocyte–endothelial adhesion is perhaps the most well-characterized role of cell surface glycoRNAs. GlycoRNAs expressed on the surface of monocytes and neutrophils promote their attachment to endothelial cells, which is a crucial prerequisite for immune surveillance and inflammation. To illustrate, Huang et al. [5] found that cell surface glycoRNAs facilitated monocyte adhesion to human umbilical vein endothelial cells in vitro, and their disruption using antisense oligos impaired these interactions [5]. Similarly, cell surface glycoRNAs present on neutrophils were found to bind P-selectin on endothelial cells and were vital for the capture and rolling of neutrophils along the vascular wall [4]. Using an in vivo mouse model of acute inflammation, Zhang et al. [4] also showed that enzymatic degradation of cell surface RNA substantially reduced neutrophil recruitment to inflammatory sites, with minimal effect on the localization of neutrophils to the peripheral blood, bone marrow, and spleen [4]. Taken together, these findings highlight a specific defect in vascular adhesion in the absence of cell surface RNA.

The adhesion function of cell surface RNAs can be attributed to their glycosylated nature. When the carbohydrate moieties of RNAs are blocked, cell adhesion is severely impaired [4]. Notably, the Siglec family of immune lectins recognizes the sialylated glycans of glycoRNAs, implicating a broader role for cell surface RNAs in immune cell signaling and checkpoint regulation [3,4]. Siglec engagement is RNase-sensitive, indicating that cell surface glycoRNAs act as ligands, and can, in turn, possibly modulate immune responses [3]. Thus, leukocyte recruitment involves not only protein-based interactions, but also cell surface RNA-mediated adhesion and signaling.

#### Regulation of immune cell activation via modifying receptor–ligand signaling

Emerging evidence supports a model in which cell surface RNAs regulate receptor–ligand interactions on the plasma membrane. Li et al. [15] reported that a subset of cell surface RNAs, called heparan sulfate-associated RNA (HepRNA), colocalizes with the poliovirus receptor (PVR/CD155) and promotes its interaction with killer cell immunoglobulin-like receptor 2DL5 (KIR2DL5), forming an immune checkpoint that ultimately suppressed NK cell cytotoxicity [15]. RNase treatment disrupted KIR2DL5 engagement without affecting other PVR ligands, and supplementation with exogenous RNA restored this interaction [15]. This data illustrates a co-receptor function of cell surface RNA in enhancing weak ligand–receptor affinities.

Further, cell surface RNAs have directly been found to modulate activation of efferocytes, such as BMDMs, and regulate their activation in response to apoptotic cells [36]. The presence of N-glycans on cell surface RNAs were found to prevent BMDM activation via innate TLR7 or TLR3 sensing by covering the modified RNA base acp^3^U, which has immunogenic properties strong enough to elicit inflammatory immune cell activation [36]. The de-N-glycosylation of cell surface RNA, disruptions in TLR7/TLR3, or genetic deletion of the DTWD2 enzyme responsible for acp^3^U production all abrogated this efferocyte activation, as shown through a reduction in Type I interferon production by BMDMs [36]. This recently published study by Graziano et al. [36] highlights the role of cell surface RNAs in modulating innate immune cell activation in response to immunogenic stimuli such as apoptotic cells, implicating them in playing a larger role in the activity and response of innate immune cells.

#### Functional modulators in cancer progression

Distinct cell surface RNA expression profiles have been discovered across various cancer types, suggesting that cell surface RNA landscapes undergo dynamic changes during oncogenic transformation and may contribute to the pathogenesis of cancer. In breast cancer, Lv et al. [6] found that cell surface RNA species associated with the ECM, a critical component of the tumor microenvironment, were up-regulated when compared with non-cancerous tissues. Many of these ECM-related genes are associated with collagens, such as *COL5A1*, *COL12A1*, and *CNN1*, which play crucial roles in the migration, invasion, and metastasis of breast cancer. In colorectal cancer, it was found that a glycosylated form of miR-27a-3p regulates oncogenic signaling by sequestering hsa_circ_0004194, thereby inhibiting the RXRα/β-catenin axis and effectively limiting cancer progression [40]. These findings support the utility of cell surface RNA profiles as biomarkers and functional modulators of cancer progression.

#### Source of autoantigens in autoimmunity

Moreover, cell surface RNA–RBP clusters have been proposed to explain the long-standing clinical finding of extracellular RBP autoantigens in autoimmune diseases, such as systemic lupus erythematosus (SLE) [3]. To date, several RNA and RNA-associated autoantigens have been characterized in autoimmune disease, but canonically, such antigens only become exposed to the extracellular space following cell death [3,41–44]. Notably, however, the Y RNA family of glycoRNAs and their binding proteins are known autoantigens in SLE, whose cell surface exposure provides a mechanism for immune recognition in the absence of cell death [3]. This model suggests that dysregulation or altered organization of cell surface RNA–RBP clusters could provide an accessible source of autoantigens and thereby contribute to the pathogenesis of autoimmune disease [45].

### Functions of cell surface RNAs on non-immune cells

#### Modulators of epithelial barrier integrity

Cell surface RNAs also affect epithelial cell barrier function. Treatment of alveolar epithelial cells with an RNase cocktail not only reduced the abundance of cell surface RNAs, but also resulted in a 30% decrease in transepithelial electrical resistance, indicating compromised barrier integrity [24]. Simultaneously, eliminating cell surface RNA reduced the number of bound or internalized influenza A virus particles, again suggesting that cell surface glycoRNAs contribute to alveolar epithelial barrier function and may serve as attachment factors during viral infection [24].

This established role of cell surface RNAs in modulating alveolar epithelial barriers [24] opens a cascade of potential roles they could play in regulating epithelial integrity throughout the body. Past studies have linked non-coding RNAs to the maintenance of tight junctions and epithelial cell layers [46,47], and it is plausible that cell surface RNAs could play similar roles. Current studies have specifically implicated non-coding RNAs, such as lncRNAs and miRNAs, in directly regulating the expression of proteins that compose tight junctions and regulate epithelial cell proliferation, survival, and apoptosis [46,48]. While cell surface RNAs are found on the exterior of cells, rather than the interior, like most ncRNAs, it is possible that they, along with their associated glycosyl groups and RBPs, can modulate junctional protein–protein interactions. They could also act as signaling molecules to educate epithelial cells about the extracellular environment, influencing expression of genes that regulate epithelial cell function and tight junctions during infection or damage. The latter would be especially important in mucosal barrier environments, such as the digestive, respiratory, and reproductive tracts, where constant surveillance of the external environment is crucial to maintain homeostasis and combat external threats such as infection-causing microbes, allergens, and physical damage [49].

#### Signaling scaffolds and coordinators of membrane organization

At the molecular level, cell surface RNAs colocalize with cell surface RBPs to form clusters that act as scaffolds for signaling and molecular exchange. Perr et al. [31] demonstrated that these cell surface RNA–RBP clusters facilitated the entry of a cell-penetrating peptide, trans-activator of transcription (TAT), supporting the idea of an RNA-dependent import system on the cell membrane. Disruption of these cell surface RNA–RBP clusters by RNase digestion inhibited TAT internalization into the cell, indicating that cell surface RNAs may play an active role in modulating membrane permeability and endocytosis via an alternative internalization pathway.

#### Potential role of cell surface RNA in neuronal communication and synaptic stability

There may lie a potential role for cell surface RNA in neuronal communication and synaptic plasticity. In general, ncRNAs are found in neural synapses and are vital for their stability, plasticity, and neuron–neuron communication. For example, lncRNAs *NEAT2/MALAT1* have been shown to regulate synapse formation [50], while *RBFOX1* has been shown to modulate neuron excitability through regulation of ion channels and neurotransmitter receptors [51]. Further, the lncRNA neuronal *HOTAIRM1* has been shown to promote spinal motor neuron differentiation, with its deletion also impairing neurite growth, resulting in impaired neurotransmission and synaptic connectivity [52]. The roles of many intracellular ncRNAs in the neural synapse have previously been described, but the role of cell surface glycoRNAs in the CNS is yet to be determined.

One study by Xin et al. [28], however, did find that human glioma cells had a high expression of cell surface RNAs and that their depletion abrogated glioma cell proliferation. Glioma tumor cells originate from glial cells in the brain, and although glial cells are not directly involved in neuronal communication, they support this communication via myelination of neurons, structural support, nutrient supply, and homeostasis of the neural environment [53]. It is possible that glial cells similarly express cell surface RNAs and that they can modulate their function in the CNS.

Cell surface RNAs on neurons may also play a direct role in synaptic plasticity. Van Oostrum et al. [54] found evidence of Sidt1/Sidt2 expression on neurons, which, as mentioned previously, are RBPs that can bind RNA and may be directly involved in translocating RNAs to the cell surface. Van Oostrum et al. [54] found that Sidt1 expression was increased during synaptic upscaling, a process in which synapses are strengthened to increase responsiveness to input signals via increasing signal receptor sensitivity [55,56]. An increase in Sidt1 expression during synaptic upscaling could directly result in increased cell surface RNA expression on neurons, indicating that they may play an important role in synaptic stability. Even further, Sidt1 and Sidt2 were shown to be decreased during synaptic downscaling [54], indicating that cell surface RNA expression may dynamically change during synaptic scaling, thus alluding to a potential role for them in synaptic plasticity and stability.

#### Potential role of cell surface RNA in cell–cell communication

Cell surface RNAs may also play a role in cell–cell communication during embryogenesis and early development. RNA–RBP complexes on the cell surface may be transient intermediates for complexes that are eventually released by the cell in EVs. These vesicles can be taken up by nearby or distant cells, where its contents are able to exert an effect in the receiving cell. Previous research has shown that during embryogenesis and early development in utero, EVs containing miRNAs are exchanged between tissues to help regulate and coordinate cell growth and progenitor cell differentiation [57].

In the same respect, some ncRNAs have been shown to be “freely” secreted by cells while bound to RBPs that protect them from degradation in the external environment [32,33]. These secreted ncRNAs can act as systemic messengers, similar to cytokines or hormones, and elicit changes in neighboring or distant cells. Some have coined these secreted ncRNAs as “RNAkines” as they can modulate gene expression and thus cellular activity, including differentiation, proliferation, and apoptosis, once they are taken up by receiving cells [34,58]. Again, cell surface RNAs may be an intermediate state of these “RNAkines” as they are similarly found to be associated with RBPs on cell surfaces, but whether they are secreted into the extracellular space needs to be examined. As mentioned earlier, a study by Morozkin et al, did find cell surface RNAs that were transiently bound to the surface of cancerous cell lines (HeLa and A431), indicating that it may be possible that they are released into the extracellular environment.

On the other hand, as mentioned earlier in our talk on epithelial barrier integrity, cell surface RNAs could act as signaling molecules on a wide array of other cells as well. Similar to other surface-bound or transmembrane receptor molecules, their presence on the cell surface places this class of RNAs in a prime location to sense changes in the extracellular space. There is potential for these cell surface RNA–RBP complexes to initiate intracellular signaling cascades and alter gene expression in response to external stimuli; however, whether they are linked to internal cellular components is yet to be fully determined. The current literature states that cell surface RNAs are attached to the surface via unknown linkers [4]; determining what these linkers are will provide more insight into their potential role as a signaling molecule.

## Impact and Applications of Cell Surface RNA

**Fig. 4.**
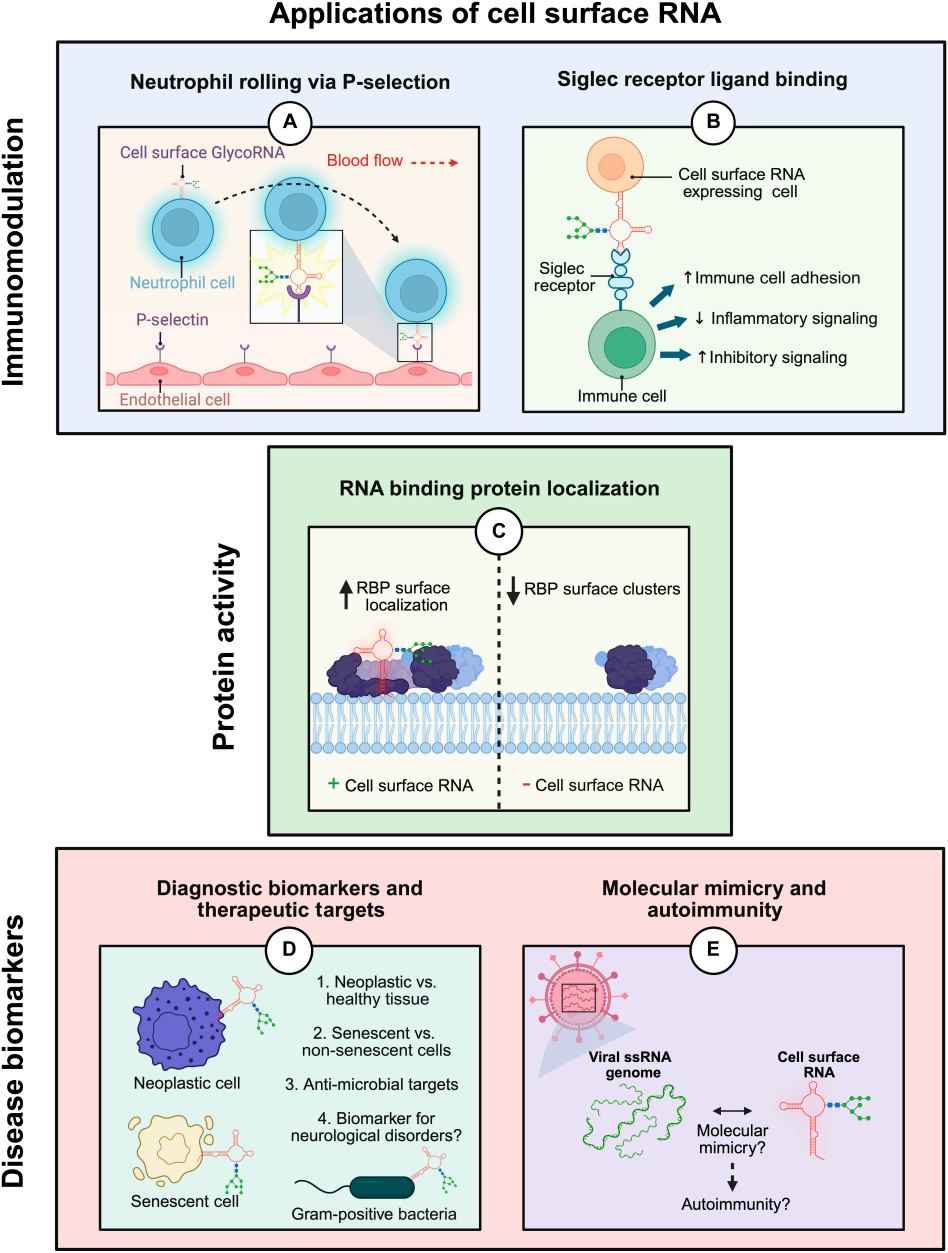
Summary of proposed applications of cell surface RNA. (A) Neutrophil rolling via interaction between cell surface RNAs and P-selectin binding promotes migration and inflammation in target tissues. (B) Siglec receptor ligand binding by cell surface RNAs can promote migration, adhesion, inflammatory signaling, and improve targeted drug delivery. (C) Cell surface RNAs directly regulate RBP localization and clustering on the cell surface, downstream regulating RBP activity. (D) Cell surface RNAs may act as diagnostic biomarkers for cancer, cell senescence, and neurological disorders, while also acting as potential target for antimicrobial therapies. (E) Viral ssRNA genomes may contain genomic elements similar to autologous cell surface RNAs, opening a new avenue to study molecular mimicry between the two, and implicating a potential role for cell surface RNA in regulating autoimmunity. Created in BioRender. Jose, P. [2026] https://BioRender.com/4gaky9k.

Cell surface RNAs represent a groundbreaking discovery in cell surface biology, combining RNA with N-glycans to form a novel class of hybrid molecules. They are reshaping our understanding of RNA biology by revealing its extracellular roles in immunity, cell communication, and disease. Their unique properties as glycosylated cell surface molecules open new avenues for diagnostics, therapeutics, and fundamental research (Fig. 4).

### Application of cell surface RNAs in immunomodulation

Cell surface RNAs play critical roles in immune regulation, with cell surface RNAs on neutrophil surfaces promoting P-selectin-mediated capture and rolling in the vasculature, which is essential for inflammatory responses [4]. Their selective binding to P-selectin, but not E-selectin, suggests a unique mechanism for leukocyte–endothelial interactions that differs from classical ligands like PSGL-1. The biological role of cell surface RNAs also extends to cell–cell communication, where they facilitate monocyte–endothelial adhesion and participate in intercellular signaling [5]. Provided the molecular mechanisms of their recognition are first better characterized, cell surface RNAs show great promise in being novel regulators of immune system responses.

Beyond neutrophils, cell surface RNAs interact with immune receptors, implicating them in immune modulation and as potential therapeutic targets for drug delivery and immunomodulation for autoimmune diseases and cancer [3,8,36]. Engineered cell surface RNAs could exploit selectin interactions to direct leukocytes to specific tissues or block pathological inflammation [3,4,8,59]. Additionally, their interactions with Siglec receptors offer opportunities for the generation of new cancer immunotherapies [3]. Antibody-based or lectin-based probes specific to disease-associated cell surface RNAs may also be used to enable selective detection of malignant or activated immune cells. Further, modulating cell surface RNA-signaling pathways could offer a novel approach to influence immune responses, clearly showcased by Graziano et al. [36], where N-glycan modified cell surface RNAs directly regulate innate immune cell activation. One study also implicated cell surface RNAs in playing a role alveolar epithelial barrier integrity, with their removal decreasing transepithelial electrical resistance, while, surprisingly, conversely reducing the number of internalized influenza virus particles [24]. This opens up a new door for the role of cell surface RNA in regulating immunity, specifically barrier immunity, as epithelial or mucosal barriers are the first line of defense for many microbial diseases.

### Application of cell surface RNAs in modulating protein activity

One of the most consistent findings in the current literature was the interaction between cell surface RNA and RBPs on the cell membrane [4,31]. Surface RBPs are important to multiple cellular functions, including cell–cell interactions, signaling, communication, and signal transduction into cells [45,60]. Studies found that surface RNA is necessary for RBP protein localization on the cell surface, which points toward a potential regulatory role of cell surface RNAs [15,31,45]. If cell surface RNAs regulate RBP protein localization, then, hypothetically, altering the amount of surface RNA could alter the amount of RBP clusters on the cell surface, thus affecting different surface RBP-mediated processes. This stabilizing interaction was found to work both ways as cell surface proteins are thought to protect cell surface RNAs from degradation, potentially allowing for prolonged extracellular activity from the latter [5].

Additionally, the presence of RNA on the cell surface is enabled by specific transporters like the Sidt RBPs, highlighting an RNA-specific trafficking mechanism to the cell surface that is distinct from EV delivery of RNA [4]. This is of importance as Sidt transporters are normally implicated in mediating dsRNA uptake and transport from endocytic compartments into the cell [26], which begs the question if glycoRNAs are able to alter their activity directly to promote extracellular RNA transport instead.

The regulatory roles that cell surface RNAs and RBPs have on one another and their implications on cellular activity is one of the most, if not the most promising avenue to uncover novel regulatory mechanisms and therapeutic targets for disease.

### Application of cell surface RNA as biomarkers for disease

The stability of cell surface RNA, combined with their cell type-specific expression, also makes them promising candidates for diagnostic biomarkers, particularly in cancer and inflammatory conditions, where altered cell surface RNA profiles may correlate with pathological progression. For example, distinct RNA profiles have been identified in colon cancer tissues, with glycosylated miR-27a-3p linked to tumor suppression [40]. Similarly, breast cancer studies using membrane-specific RNA extraction (MREMB) have identified disease-associated cell surface glycoRNAs [6]. These include the cell surface-enriched lncRNA TALAM1 and ECM-related COL5A1 mRNA that are consistently overexpressed in breast cancer cells compared to non-neoplastic breast epithelial cells [6]. These cell surface RNAs, localized to the cell surface and secreted compartments, represent promising candidates for non-invasive diagnostics due to their detectability in membrane fractions and their association with multiple breast cancer subtypes [6]. Although the exact function of cell surface RNA is only newly being discovered, it is quite clear that they can act as biomarkers for cancer stage and metastasis state. Whether or not they play a direct role in tumorigenesis or anti-tumor immunity needs to be determined going forward, as well as whether there are cancer-specific cell surface RNAs that differ from normal tissue or even between cancer types.

Similarly, non-coding RNAs have been established as prognostic and diagnostic biomarkers in neurological disorders related to motor neuron function, such as amyotrophic lateral sclerosis and spinal muscular atrophy [61], as well as being implicated in regulating the overall activity of spinal motor neurons [52]. The role of cell surface RNAs, together with their associated cell surface RBPs, in the central nervous system has not been explored, and thus, whether they can act as biomarkers for neurological disorders or regulate neuron function is still unknown, yet opens many new avenues to study cell surface RNA function in the body.

There may also lie a potential role for cell surface RNA in detecting cellular aging or senescence as their expression was found on cancerous cells after long-term culturing [10]. As cells age, their function is impaired due to the inability to properly replicate DNA and divide, resulting in the accumulation of waste molecules and DNA damage within the cells [62]. Detection of cell surface RNA on non-senescent and senescent cells will further validate whether their expression can be linked as a biomarker of senescence.

Researchers have previously employed the use of TLR7 to detect cell surface RNAs [15], a toll-like receptor that is involved in detecting ssRNA viruses like SARS-CoV-2. There is a possibility that some ssRNA viral genomes contain genomic elements similar to autologous cell surface RNAs on human cells, opening a new avenue to study molecular mimicry between the two [63], and implicating a potential role for cell surface RNA in regulating autoimmunity. In the context of bacterial infection, gram-positive bacteria-specific surface RNAs present as new potential targets for antimicrobial therapies [11,12,30]. However, the above will not be possible without first generating a standard protocol for cell surface RNA isolation and sequencing.

Finally, HS has also been implicated in cell surface RNA presentation, where they found that it was necessary for stable presentation of both RNA and their associated RBP on the cell surface [15,60]. These results were obtained using different cancer cell lines, raising multiple questions such as the following: (a) What effect does cell surface HepRNA have on the immune system? (b) Is there differential expression of cell surface HepRNA on cancer cells vs normal tissue? and (c) Is there a link between cell surface HepRNA and cancer cells based on metastasis state, malignancy state, or cancer cell type?

## Conclusion

The discovery of cell surface RNAs, particularly cell surface glycoRNAs, has changed the understanding of RNA biology by revealing its dynamic presence beyond the intracellular world. RNA was long believed to function exclusively within the confines of the cell; however, recent breakthroughs have revealed its presence on the cell surface as a membrane-associated molecule, where it plays pivotal roles in immune activation, cellular adhesion, signal transduction, disease mechanisms, and diagnostic cancer biomarkers. Their unique properties as glycosylated cell surface molecules open new avenues for diagnostics, therapeutics, and fundamental research. However, challenges remain, including the need for improved detection methods. Current tools such as ARPLA and HRP-based proximity labeling are practical, but lack the throughput required for clinical application [14].

Advances in cell surface RNA detection and isolation techniques, such as metabolic labeling [3,24], PLA [14], and TLR7-Fc fusion proteins [15], have begun to elucidate their spatial organization and mechanistic roles. Key findings from these methods highlight that cell surface RNAs facilitate leukocyte–endothelial interactions, modulate immune responses through interactions with Siglec receptors and innate pattern recognition receptors [4,36], and contribute to epithelial barrier integrity. Additionally, they serve as co-receptors in ligand–receptor signaling and organize membrane-associated RNA–protein complexes that may influence autoimmunity and cancer progression [15,31,45].

Notable challenges remain in understanding cell surface RNA biology. The precise mechanisms governing their biogenesis, transport, and membrane anchoring are still unclear. Additionally, their functional roles in non-immune cells and potential applications in diagnostics and therapeutics largely remain unexplored. Moving forward, research efforts should prioritize 3 key areas: (a) elucidating biosynthetic pathways by identifying the enzymes and molecular machinery responsible for cell surface glycoRNA modification and membrane localization; (b) developing innovative detection tools, including multiplexed and high-throughput imaging techniques to study cell surface RNA dynamics in real time; and (c) investigating therapeutic potential by evaluating cell surface RNAs as novel targets for immunotherapy, particularly in cancer and autoimmune diseases.
